# E3 ubiquitin ligases: key regulators of osteogenesis and potential therapeutic targets for bone disorders

**DOI:** 10.3389/fcell.2024.1447093

**Published:** 2024-08-15

**Authors:** Heng-Rui Zhang, Yang-Hao Wang, Zhen-Ping Xiao, Guang Yang, Yun-Rong Xu, Zai-Tian Huang, Wei-Zhou Wang, Fei He

**Affiliations:** ^1^ School of Medicine, Kunming University of Science and Technology, Kunming, Yunnan, China; ^2^ Department of Orthopedic, Qujing Affiliated Hospital of Kunming Medical University, Qujing, Yunnan, China; ^3^ Department of Pathology, The First Affiliated Hospital of Kunming Medical University, Kunming, Yunnan, China; ^4^ Department of Orthopedics, The First Affiliated Hospital of Kunming Medical University, Kunming, Yunnan, China; ^5^ Department of Pain and Rehabilitation, The Second Affiliated Hospital of University of South China, Hengyang, Hunan, China; ^6^ Department of Trauma Surgery, The First Affiliated Hospital of Kunming Medical University, Kunming, Yunnan, China

**Keywords:** E3 ubiquitin ligases, osteogenesis, bone-related diseases, osteoblast differentiation, therapeutic targets

## Abstract

Ubiquitination is a crucial post-translational modification of proteins that mediates the degradation or functional regulation of specific proteins. This process participates in various biological processes such as cell growth, development, and signal transduction. E3 ubiquitin ligases play both positive and negative regulatory roles in osteogenesis and differentiation by ubiquitination-mediated degradation or stabilization of transcription factors, signaling molecules, and cytoskeletal proteins. These activities affect the proliferation, differentiation, survival, and bone formation of osteoblasts (OBs). In recent years, advances in genomics, transcriptomics, and proteomics have led to a deeper understanding of the classification, function, and mechanisms of action of E3 ubiquitin ligases. This understanding provides new insights and approaches for revealing the molecular regulatory mechanisms of bone formation and identifying therapeutic targets for bone metabolic diseases. This review discusses the research progress and significance of the positive and negative regulatory roles and mechanisms of E3 ubiquitin ligases in the process of osteogenic differentiation. Additionally, the review highlights the role of E3 ubiquitin ligases in bone-related diseases. A thorough understanding of the role and mechanisms of E3 ubiquitin ligases in osteogenic differentiation could provide promising therapeutic targets for bone tissue engineering based on stem cells.

## 1 Introduction

**FIGURE 1:**
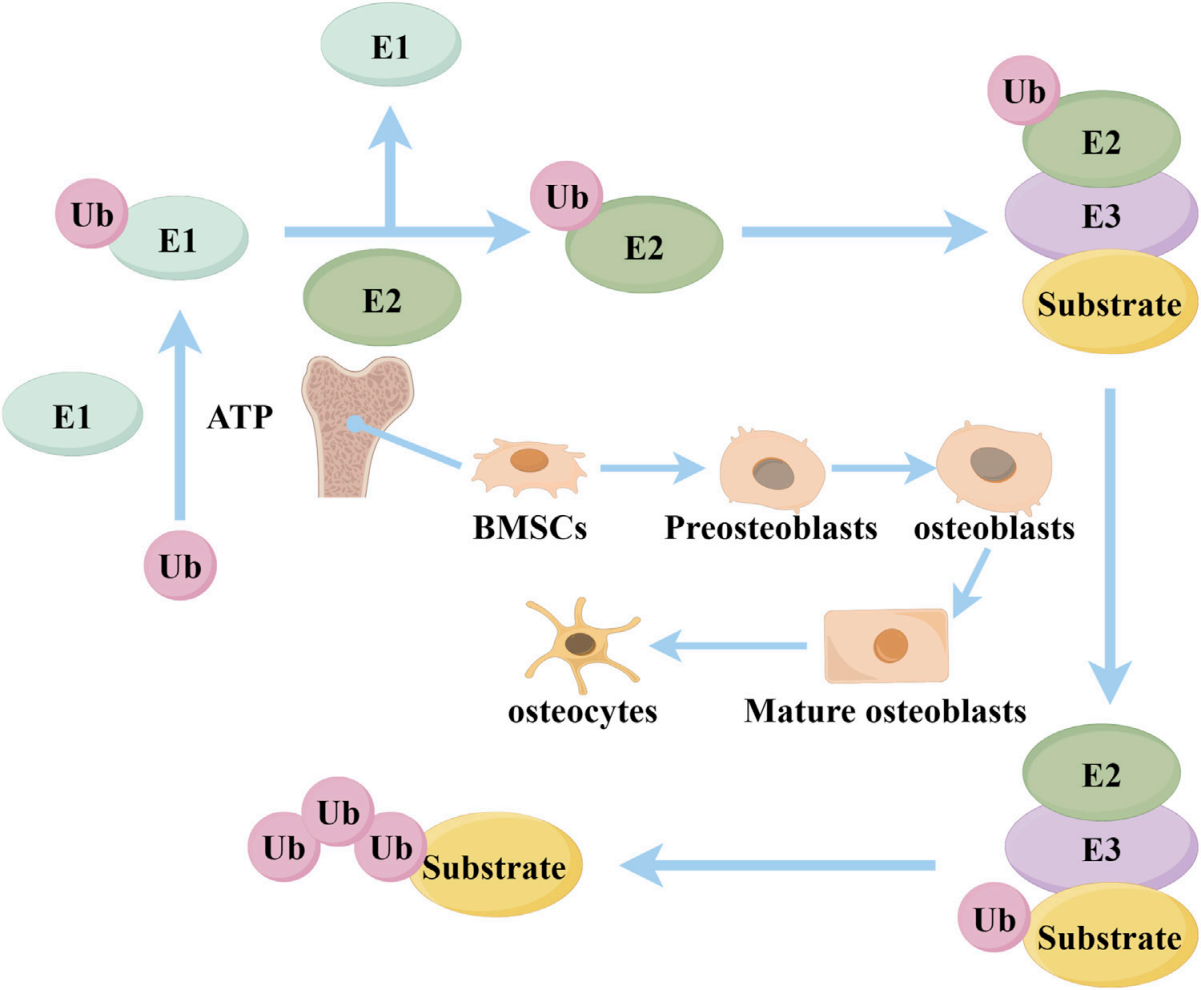
The covalent interaction between a ubiquitin molecule and the ubiquitin-activating enzyme E1 occurs through the energy of ATP. Ubiquitin (Ubs) can then be transported to the ubiquitin-conjugating enzyme E2, which can form a complex with the E3 ubiquitin ligase and substrate. Subsequent to this, the Ubs are transported to E3, where successive Ub molecules are supplied to form a multimeric Ub chain (By Figdraw).

Ubiquitination is an important post-translational modification mechanism within cells, involving the covalent binding of ubiquitin molecules to substrate proteins, affecting the stability, activity, and function of proteins (Suryadinata et al., 2014; Müller et al., 2021). The ubiquitination process relies on the coordinated action of three types of enzymes: E1 ubiquitin-activating enzymes, E2 ubiquitin-conjugating enzymes, and E3 ubiquitin ligases. E1 enzymes first activate ubiquitin through an ATP-dependent mechanism, then E2 enzymes receive the ubiquitin, and finally, E3 enzymes specifically recognize substrate proteins and transfer ubiquitin from E2 to the substrate, forming mono-ubiquitination or polyubiquitin chains (Toma-Fukai and Shimizu, 2021) (Figure 1). E3 ubiquitin ligases, as the core enzymes in the ubiquitination process, play a crucial role (Foot et al., 2017). E3 ligases are mainly divided into three major families: the RING domain family, the HECT domain family, and the RBR type family (Uchida and Kitagawa, 2016). The RING domain family contains a RING finger domain and transfers ubiquitin directly to the target protein by binding with the E2 enzyme (Lou et al., 2023). The HECT domain family contains a HECT domain; the E2 enzyme first transfers ubiquitin to the HECT enzyme, which then transfers it to the target protein (Takeda et al., 2023). The RBR type family has two RING domains connected by a BR domain, possessing functions of both RING and HECT types (Pao et al., 2018). Among them, the TRIM family is an important subclass with unique RING, B-box, and coiled-coil domains (Cheng et al., 2024). Studies have shown that more than 600 kinds of E3 ubiquitin ligases are expressed in the human body, with various E3 ligases mediating osteogenesis and osteogenic differentiation through different pathways (Huang et al., 2024).

As a crucial component of the human body, bone tissue performs essential functions such as providing structural support, protecting internal organs, hematopoiesis, and mineral storage. The formation and maintenance of bone tissue involve a dynamic balance process, entailing the interaction of numerous cell types and signaling molecules (Berendsen and Olsen, 2015). Osteogenic differentiation, the core process of bone formation, encompasses the complex transformation of bone marrow mesenchymal stem cells (BMSCs) into osteoprogenitor cells, pre-osteoblasts, mature osteocytes, and eventually OBs. This process involves multiple intracellular and intercellular signaling pathways, transcription factors, growth factors, and microRNAs (miRNAs) that work together to create a comprehensive regulatory feedback mechanism for bone metabolism (Liu C et al., 2022; Li et al., 2023). During osteogenic differentiation, E3 ubiquitin ligases regulate the differentiation and function of osteoblasts by ubiquitinating target proteins, thereby altering their stability, activity, or interactions. Specifically, E3 ubiquitin ligases play both positive and negative regulatory roles in osteogenic differentiation. This review summarizes the roles of E3 ubiquitin ligases in regulating key transcription factors and signaling pathways in osteogenic differentiation. It provides a detailed analysis of the mechanisms of osteogenic differentiation and offers new perspectives for the research and treatment of bone-related diseases. Future research will elucidate the regulatory mechanisms of E3 ubiquitin ligases and explore their potential therapeutic strategies in clinical applications.

## 2 Regulatory mechanisms of E3 ubiquitin ligases during osteogenic differentiation

This review summarizes and analyzes research on E3 ubiquitin ligases in osteogenic differentiation, finding that E3 ubiquitin ligases have both positive and negative regulatory roles in the differentiation and functional regulation of OBs. Specifically, some E3 ubiquitin ligases regulate the degradation of key regulatory proteins through ubiquitination, inhibiting the differentiation and function of OBs. Conversely, other E3 ubiquitin ligases enhance the stability and activity of key regulatory proteins through ubiquitination, promoting the differentiation and function of OBs. This review will detail six classes of E3 ubiquitin ligases that negatively regulate osteogenic differentiation and five that positively regulate the osteogenic differentiation process.

### 2.1 Negative regulation

#### 2.1.1 RNF185

RNF185 (Ring Finger Protein 185) is a member of the RING domain family, an E3 ubiquitin ligase that can interact with key proteins of the Wnt pathway, regulating their stability and function. In 2011, Tang et al. first discovered RNF185 as an E3 ubiquitin ligase on the mitochondrial outer membrane (MOM), regulating selective autophagy through interaction with BNIP1, which is co-localized with it in mitochondria (Tang et al., 2011). In 2013, studies revealed RNF185 as a novel E3 ubiquitin ligase in the endoplasmic reticulum-associated degradation (ERAD) pathway, targeting the cystic fibrosis transmembrane conductance regulator (CFTR) for degradation in a RING domain and proteasome-dependent manner (El Khouri et al., 2013). Earlier studies have shown that RNF185 plays a role in both proteasome and autophagy-mediated degradation processes (Kraft et al., 2010). In 2014, Zhou et al. utilized qRT-PCR and alkaline phosphatase (ALP) activity assays and discovered that RNF185 inhibits the Wnt signaling pathway by promoting the degradation of Dvl2 (dishevelled-2), preventing the stabilization and accumulation of β-Catenin, thereby inhibiting osteogenic differentiation in mouse cranial-derived MC3T3-E1 cells (Zhou et al., 2014). Conversely, in 2022, Pan et al. discovered that RNF146, homologous to RNF185, can enhance the Wnt pathway. Mice lacking RNF146 suffered from severe osteopenia due to defects in OB production and subsequent impaired osteocalcin production, along with glucose intolerance (Matsumoto et al., 2017). As a negative regulator, RNF185 may play a significant role in bone-related diseases. Further research into the relationship between RNF185 and bone-related diseases could reveal its role in the development and progression of these diseases. Moreover, studying the regulatory mechanisms and downstream targets of RNF185 may provide new insights for developing novel targets, drugs, and therapeutic strategies for treating bone-related diseases.

#### 2.1.2 Trim21

**FIGURE 2:**
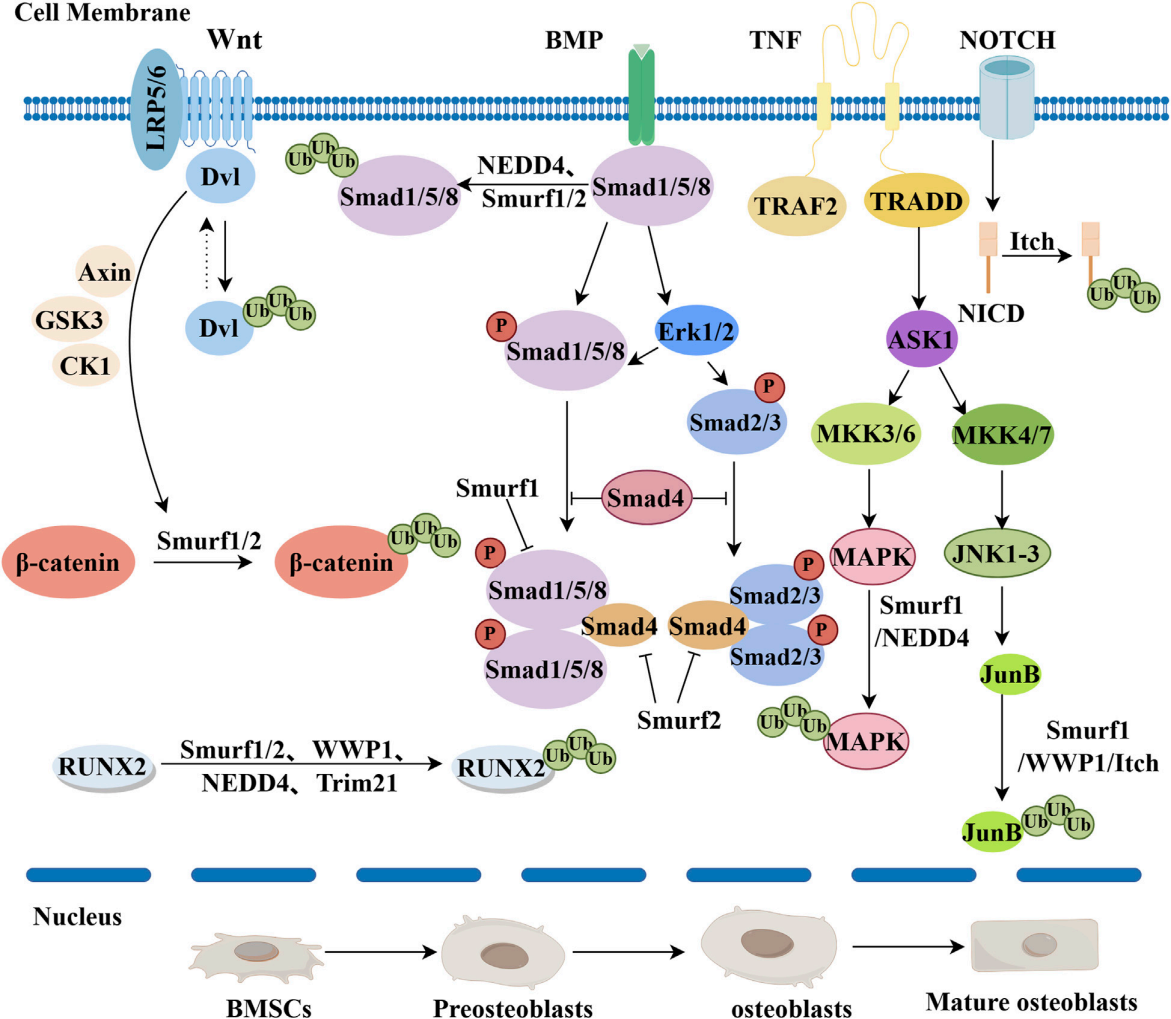
The mechanisms of negative regulation of E3 ubiquitin ligases during osteogenic differentiation are as follows. The Wnt/B-catenin pathway: RNF185 blocks the Wnt pathway by degrading Dishevelled 2 (Dvl2). Smurf1/2 ubiquitinates β-catenin to exert an inhibitory effect. Finally, β-catenin is catabolized. BMP pathway: Smurf1 and NEDD4 target Smad1/5/8, leading to its ubiquitination and degradation. Smurf2 can also target Smad1 for ubiquitination, similar to Smurf1, negatively regulating BMP signaling. TNF pathway: Smurf1 inhibits the TNF pathway by targeting mitogen-activated protein kinases (MAPKs) and JunB. WWP1 and Itch inhibit the TNF pathway by targeting JunB. Notch pathway: Itch binds NICD, promotes ubiquitination and degradation of NICD, and negatively regulates Notch signaling. RUNX2 ubiquitination: Smurf1/2, WWP1, NEDD4, and Trim21 all ubiquitinate RUNX2, thereby inhibiting osteogenic differentiation (By Figdraw).

E3 ubiquitin ligase Trim21 is a member of the TRIM family, a group of proteins characterized by common structural features, including a RING finger domain, a B-box domain, and a coiled-coil domain (Li et al., 2014). Trim21 plays a critical role in the differentiation of OBs and is broadly involved in physiological and pathological processes in various cells through the ubiquitination of key proteins. In their 2020 study, Si et al. first discovered that Trim21 negatively regulates the expression of Runx2 through interaction with the SET domain of lysine methyltransferase 7/9 (SET7/9) (Si et al., 2020). Concurrently, Xian et al. noted that Trim21 inhibits osteogenic differentiation of mesenchymal stem cells by promoting the k48 ubiquitin-mediated degradation of Akt (Xian et al., 2022). In this process, Trim21 tags Akt for degradation by attaching ubiquitin molecules to specific sites on the Akt protein, known as k48 linkage (Wang et al., 2023). This ubiquitination modification leads to a pathway where Akt is degraded, thus reducing its activity and stability. By promoting the degradation of Akt, Trim21 is able to inhibit the differentiation capability of mesenchymal stem cells into osteocytes (Xian et al., 2022). Additionally, studies have found that the loss of TRIM21 activates the YAP1 and β-catenin signaling pathways, enhancing the stability and activity of YAP1 and β-catenin, thereby promoting the proliferation and differentiation of osteoblasts, which increases bone formation (Liu R-X et al., 2023). Recent research has found that the deficiency of Trim21 can promote bone formation and osteogenic differentiation by increasing the expression levels of osteogenic genes such as Runx2 (Figure 2) and Osterix in mouse BMSCs, cranial OBs, and pre-osteoblastic MC3T3-E1 cell lines (Liu R-X et al., 2023). This discovery is significant for our understanding of the regulatory mechanisms of stem cell differentiation and bone tissue formation and repair. However, the role of Trim21 in regulating normal bone homeostasis remains unclear and may lead to degenerative bone diseases such as osteoporosis.

#### 2.1.3 WWP1

WWP1 belongs to the E3 ubiquitin ligase C2-WW-HECT family. The C-terminal of WWP1 contains a HECT domain, which can interact with ubiquitin-conjugating enzymes UbcH5 or UbcH7 (Zhi and Chen, 2012). WWP1 and Schnurri-3 (Shn3) proteins are negative regulators of OBs function, and inhibition of WWP1 may promote bone deposition in the treatment of osteoporosis (Tucker et al., 2018). WWP1 can promote the degradation of Runx2, thereby hindering the differentiation process of osteoprogenitor cells into mature OBs (Shu et al., 2013) (Figure 2). Researchers have found through further studies using the Shn3 (−/−) mouse model that WWP1 mediates the degradation of Runx2 by binding to Shn3. Simultaneously, Shn3 promotes the ubiquitination and degradation of Runx2 protein by recruiting WWP1 to Runx2 (Jones et al., 2006). Subsequently, Researchers have noted that the JunB protein is a key transcription factor regulating the differentiation of MSCs into OBs. WWP1 targets JunB for ubiquitination and degradation after prolonged exposure to tumor necrosis factor (Figure 2) (Zhao et al., 2010; Zhao L et al., 2011). Additionally, research by Shu and others points out that the migration of OBs is crucial for bone remodeling and repair processes. WWP1 can also inhibit the migration of OBs by promoting the degradation of the key protein focal adhesion kinase (FAK), thereby impairing the OBs migratory ability and affecting the bone remodeling process (Shu et al., 2013). Recent studies have found that exosomes from mesenchymal stem cells, containing miR-19b, inhibit the expression of WWP1 and Smurf2. This inhibition enhances the expression of the target protein KLF5 through the Wnt/β-Catenin signaling pathway, thereby promoting fracture healing (Huang et al., 2021). In summary, when WWP1 protein is activated, it can degrade key proteins related to osteogenic differentiation through the ubiquitin-proteasome system, leading to reduced expression and activity of these proteins, ultimately inhibiting osteogenic differentiation. Different types of ubiquitination have their physiological functions, but further research is needed to determine the type of ubiquitin linkage catalyzed by WWP1.

#### 2.1.4 Smurf1/Smurf2

Smurf1/Smurf2 belong to the C2-WW-HECT domain family of E3 ubiquitin ligases. They play a key role in embryonic development and adult bone homeostasis by modulating bone morphogenetic proteins, Wnt, and non-Smad signaling pathways (Lu et al., 2011). Regulation of the bone morphogenetic protein (BMP) signaling pathway involves Smurf1 and Smurf2 mediating the degradation of downstream Smad1/5/8, thereby inhibiting the BMP receptors (Murakami and Etlinger, 2019; Kushioka et al., 2020). Smurf1/Smurf2 inhibit BMP receptors by mediating the degradation of downstream Smad1/5/8 (Zhao et al., 2003; Zhang et al., 2017; Kushioka et al., 2020). As the most extensively studied members of the C2-WW-HECT E3 ligase family, they capture Smad1/5/8 via the WW domain, inhibiting BMP/Smad signal transduction and thus suppressing the osteogenic differentiation of bone marrow stromal cells (BMSCs) (Zhao et al., 2003; Zhang et al., 2017; Kushioka et al., 2020). Regarding the Wnt signaling pathway, Smurf1 and Smurf2 inhibit the pathway by reducing β-catenin levels, which further affects bone formation and bone homeostasis (Boonanantanasarn et al., 2015). Studies have also revealed the role of Smurf1 in non-Smad pathways, closely related to the Smurf1-mediated degradation of MAPKs and JUNB. Smurf1 binds to MAPKs (ERK1/2, p38 MAPK, and JNK) and promotes their ubiquitination and degradation, reducing their activity, thereby inhibiting osteogenic signaling and negatively regulating osteogenic differentiation (Lin et al., 2017; Sun et al., 2018). Additionally, Smurf1 attaches ubiquitin molecules to JUNB, marking it for proteasomal degradation. By degrading JUNB, it inhibits JUNB-mediated transcriptional activity, thus affecting the differentiation and function of osteoblasts (Zhao et al., 2010).Transcription factors such as c-JUN and RUNX2 bind to the Smurf1 promoter, in turn causing RUNX2 degradation, thus inhibiting osteogenic differentiation (Lee et al., 2014; Sun et al., 2018). Additionally, research has found that besides mediating BMP receptors, Smurf1/Smurf2 also mediate the degradation of Runx2, thereby inhibiting the differentiation of OBs (Zhao et al., 2003; Liu et al., 2020). Through the E3 ubiquitin ligases Smurf1/Smurf2, tumor necrosis factor (TNF) regulates the degradation of the OB-specific transcription factor Runx2, thereby inhibiting OB differentiation (Kaneki et al., 2006). At the same time, the action of Smurf1/Smurf2 also promotes the upregulation of Runx2 protein expression and the osteogenic differentiation of BMSCs, thereby increasing bone formation *in vivo* (Choi et al., 2014; Glimcher et al., 2007). Finally, Smurf1/Smurf2 can also be influenced by miRNAs, which mediate osteogenic differentiation by inhibiting the expression of Smurf1/Smurf2. Researchers have successfully demonstrated through bioinformatics studies and luciferase activity that the negative regulator in osteogenesis, Smurf1, is a direct target of mir-503, and that miR-503 mediates osteogenic differentiation by inhibiting Smurf1 expression (Sun et al., 2017). Recent studies have found that Smurf1 is sumoylated primarily at the C-terminal lysine residue (K324), which enhances its activity, promoting ALK2 proteolysis and the subsequent inhibition of the bone morphogenetic protein (BMP) signaling pathway (Chen et al., 2024). Additionally, recent research using bioinformatics analysis and dual-luciferase reporter assays has revealed Smurf2 as a molecular target of miR-20a. miR-20a promotes osteogenic differentiation by targeting Smurf2, while upregulating the expression level of Smurf2, thereby inhibiting osteogenic differentiation (Pang et al., 2023). Finally, new research has discovered that OTUB1 enhances the activity of the FGFR2 signaling pathway by stabilizing FGFR2 and preventing its degradation. This stability promotes the differentiation of OBs, ultimately enhancing bone formation (Zhu et al., 2023). Smurf1 and Smurf2 are crucial regulators of osteogenesis, primarily exerting a negative impact. They inhibit bone differentiation and function by suppressing BMP signaling and promoting the degradation of critical osteogenic transcription factors like RUNX2. Through the degradation of Smad proteins and modulation of Wnt and non-Smad pathways, these ligases reduce osteogenic signaling. Moreover, miRNAs and post-translational modifications further regulate their activity, either enhancing or inhibiting osteogenesis. Understanding these mechanisms offers insights into bone disease development and potential therapeutic targets for treatment.

#### 2.1.5 Itch

Itch, an E3 ubiquitin ligase in the HECT family, affects various cellular functions by regulating key signaling pathway proteins and transcription factors. Studies using Itch (−/−) mouse models have found that Itch negatively regulates osteogenic differentiation by modulating the proteasomal degradation of the positive osteoblast regulatory factor JunB protein, thereby negatively regulating the differentiation of bone marrow mesenchymal precursor cells into OBs (Zhang and Xing, 2013a). The degradation of JunB can affect the activity of the Wnt signaling pathway and reduce the activation of the BMP signaling pathway, thereby inhibiting osteogenic differentiation. Itch deficiency leads to reduced differentiation of BMSCs into OBs, resulting in decreased bone mass in mice (Zhu et al., 2017). Studies have found that Itch ubiquitinates JunB protein, marking it for proteasomal degradation. This degradation reduces intracellular levels of JunB, thereby inhibiting JunB’s promotion of osteogenesis-related genes and negatively regulating the osteogenic differentiation process (Zhang and Xing, 2013b). Itch can also ubiquitinate the phosphorylated form of dvl and promote its degradation via the proteasome pathway, inhibiting the canonical Wnt signaling and thus the osteogenic differentiation process (Wei et al., 2012). Smad1 is a crucial component in the bone morphogenetic protein (BMP) signaling pathway, which regulates the proliferation and differentiation of osteoblasts. Additionally, Itch has been found to promote the ubiquitination and degradation of Smad1, negatively regulating the osteogenic differentiation process (Liu et al., 2017). Subsequent studies have shown that knockout of the CIRC-Itch gene can inhibit the activity of alkaline phosphatase (ALP), the formation of mineralized nodules, and the expression of factors associated with stunted growth such as Runt-related transcription factor 2 (RUNX2), osteopontin (OPN), and osteocalcin (OCN) during the osteogenic induction process (Zhong et al., 2021). In summary, Itch negatively regulates the osteogenic differentiation process by modulating key signaling pathways and transcription factors such as the Wnt/β-catenin and BMP signaling pathways. However, the role of Itch in cellular signaling and regulation is highly complex, and there may be additional mechanisms and factors affecting osteogenic differentiation. Further research could reveal the detailed mechanisms of Itch in osteogenic differentiation, including its interactions with other regulatory factors and signaling pathways, as well as its role in bone-related diseases.

#### 2.1.6 NEDD4

NEDD4 is a protein containing multiple domains, including a C2 domain, several WW domains, and a HECT domain (Persaud et al., 2011). As a crucial E3 ubiquitin ligase, NEDD4 is involved in the regulation of various cellular processes, including cell proliferation, differentiation, and apoptosis (Li Y et al., 2015). Zhou et al. first discovered that lncRNA-HIF-1α and metastasis associated lung adenocarcinoma transcript 1 (MALAT1) can promote the osteogenic differentiation of BMSCs (Zhou et al., 2015). Subsequent research indicated that lncRNA SNHG1 inhibits the osteogenic differentiation of BMSCs through Nedd4-mediated ubiquitination and negative regulation of the p38MAPK signaling pathway (Jiang Y et al., 2019). Simultaneously, the E3 ubiquitin ligase NEDD4 can inhibit osteogenic differentiation by promoting the ubiquitination and downregulation of Runx2. Further research has shown that the transcriptional activation by GATA Binding Protein 4 (GATA4) can promote the osteogenic differentiation of BMSCs *in vitro* and bone formation in ovariectomized (OVX) mice through MALAT1, demonstrating that overexpression of GATA4 enhances Runx1 by interacting with the KHSRP/NEDD4 signaling axis, thus facilitating osteogenic differentiation of BMSCs (Huang X et al., 2023). In summary, as an E3 ubiquitin ligase, NEDD4 inhibits osteogenic differentiation of BMSCs by promoting ubiquitination and downregulation of Runx2. Additionally, lncRNA SNHG1 negatively regulates osteogenic differentiation through NEDD4-mediated ubiquitination, affecting the p38MAPK signaling pathway. Conversely, GATA4 transcriptional activation enhances osteogenic differentiation via the KHSRP/NEDD4 signaling axis, promoting bone formation *in vitro* and in OVX mice. Moreover, research has demonstrated that Nedd4 plays a primary role in intramembranous ossification following the differentiation of OBs, and it is crucial in bone development after mesenchymal stem cells differentiate into OBs or chondrocytic lineages (Wiszniak et al., 2016).

### 2.2 Positive regulation

#### 2.2.1 RNF146

**FIGURE 3:**
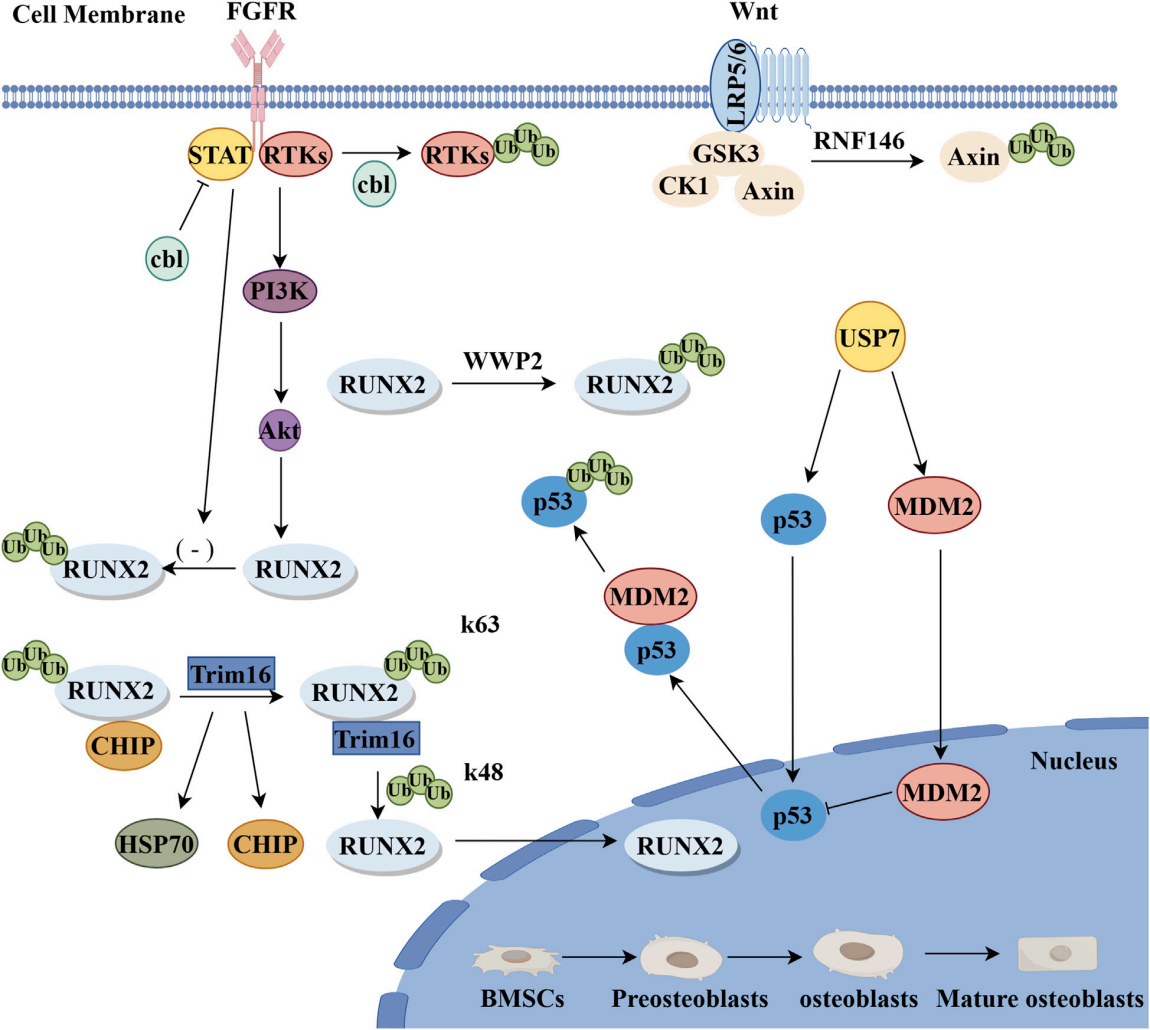
Positive regulatory mechanisms of E3 ubiquitin ligase during osteogenic differentiation. FGF pathway: cbl-mediated ubiquitination reduces the degradation of transcription factor STAT5, and there is a positive interaction between STAT5 and Runx2, which promotes the differentiation of OBs.cbl proteins promote the proliferation and differentiation of OBs through the ubiquitination and degradation of RTKs and other cbl-targeted proteins. Wnt pathway: RNF146 stimulates the Wnt pathway through the degradation of Axin to promote the proliferation and differentiation of OBs. RUNX2: RUNX2 acts as a terminal product and Trim16 promotes osteogenic differentiation by reducing CHIP-mediated ubiquitination degradation of the k48 chain of RUNX2 protein (By Figdraw).

The E3 ubiquitin ligase RNF146 is an essential ubiquitin ligase with multiple regulatory functions within the cell. RNF146 is a PAR-dependent E3 ubiquitin ligase with a ring-like domain, responsible for catalyzing the binding of ubiquitin to target proteins, thereby marking them for degradation (Nie et al., 2020). AXIN1, a key molecule in the Wnt signaling pathway, is involved in the regulation of β-Catenin activation. As a core component of the Wnt pathway, β-Catenin’s activation is crucial for the proliferation and differentiation of osteoblasts (Matsumoto et al., 2017; Xie et al., 2022). Zhang and colleagues discovered that the E3 ubiquitin ligase RNF146 controls the Wnt/β-catenin pathway through the ubiquitination of its substrate, AXIN (Zeng et al., 1997; Zhang et al., 2011). With the binding and activation of RNF146, AXIN is ubiquitinated and subsequently degraded through a proteasome-mediated process. RNF146 promotes osteogenic differentiation by inhibiting AXIN, thus enhancing the Wnt/β-catenin signaling pathway (Zhang et al., 2011) (Figure 3). The absence of RNF146 increases AXIN1 stability, impairing Wnt3a-induced β-Catenin activation and reducing FGF18 expression in OBs, which can induce the expression of the PDZ-binding motif (TAZ) (Matsumoto et al., 2017). TAZ, a transcriptional regulator of mesenchymal stem cell differentiation, is relocated to the nucleus by FGF2, activating Runx2-mediated gene transcription, stimulating osteogenic differentiation, and inhibiting adipogenic differentiation (Byun et al., 2014). Additionally, the absence of RNF146 affects the activation of the Wnt signaling pathway and reduces FGF18 expression in OBs, negatively impacting their proliferation and differentiation. Liu and colleagues, using a parabiosis mouse model, further discovered that the ubiquitin ligase RNF146, derived from apoptotic bodies, and miR-328-3p inhibit Axin1, thus activating the Wnt/β-catenin pathway (Liu et al., 2018). In summary, RNF146 can degrade Axin, leading to impaired activation of β-catenin induced by Wnt3a, reducing FGF18 expression in OBs, thus inhibiting osteogenic differentiation. Recent research has used adenoviral transduction to introduce RNF146 into BMSCs, resulting in functional Apoev-RNF146. Compared to natural Apoev, these engineered cells significantly promote osteogenesis and alleviate osteoporosis, showing great promise for the treatment of osteoporosis (Gui et al., 2024). The regulatory role of RNF146 in osteogenic differentiation represents a promising area of research. However, further studies are required to fully understand the specific roles and regulatory mechanisms of RNF146 in osteogenic differentiation.

#### 2.2.2 Trim16

Trim16 is a member of the TRIM protein family, characterized by a conserved domain known as the TRIM domain. This domain endows Trim16 with unique functions and interaction capabilities (Bell et al., 2012; Koliopoulos et al., 2016). Chen et al. discovered the regulatory roles of human galectin-3 and Trim16 in the osteogenic differentiation of BMSCs for the first time by knocking out the Galectin-3 or Trim16 genes, leading to decreased alkaline phosphatase (ALP) activity, reduced calcium deposition, lowered expression of osteogenic markers, and suppressed autophagy in osteo-induced BMSCs (Chen et al., 2020). Conversely, the overexpression of Galectin-3 and Trim16 promotes osteogenic differentiation in BMSCs. Additionally, recent studies have shown that Trim16 promotes osteogenic differentiation by stabilizing RUNX2 protein levels through reducing CHIP-mediated K48-linked ubiquitination and degradation of RUNX2 protein (Figure 3) (Zhao et al., 2021). Trim16 primarily promotes osteogenic differentiation, while the previously mentioned Trim21 acts as a bidirectional regulator involved in both the Wnt/β-catenin and YAP1/β-catenin signaling pathways, affecting both osteogenic and osteoclastic differentiation. During osteogenic differentiation, Trim16 may interact with other regulatory factors to modulate the proliferation and differentiation of OBs, though the detailed mechanisms require further investigation.

#### 2.2.3 Cbl

Casitas B-cell lymphoma (Cbl, also known as c-Cbl) protein is a RING domain-type E3 ubiquitin ligase and a key regulatory factor in signal transduction and cellular functions (Mohapatra et al., 2013). Initial studies found that the Cbl mutant (G306E) specifically inhibits the interaction between Cbl and receptor tyrosine kinases (RTKs), demonstrating that Cbl promotes the expression of OBs markers and enhances the osteogenic differentiation of cloned bone marrow-derived human mesenchymal stromal cells (hMSCs) and primary hMSCs (Sévère et al., 2011). Concurrently, studying the interaction between the Cbl mutant Y737F and PI3K/c-Cbl revealed that its inhibition increases osteogenesis *in vivo*, suggesting that eliminating the c-Cbl-PI3K interaction leads to increased bone formation (Brennan et al., 2011). Additionally, the expression of c-Cbl in hMSCs promotes OBs differentiation and osteogenesis *in vitro*. Further research indicates that Cbl-mediated ubiquitination reduces the degradation of the transcription factor STAT5, which positively interacts with Runx2, thus promoting OBs differentiation.Besides Cbl, Cbl-b also participates in controlling OBs functions. Cbl-b may regulate OBs differentiation at the post-transcriptional level, as confirmed by *in vitro* experiments (Salingcarnboriboon et al., 2010). Therefore, Cbl protein promotes the proliferation and differentiation of OBs through the ubiquitination and degradation of RTKs and other Cbl-targeted proteins (Figure 3). Recent studies have indicated that cortactin, as a cytoskeletal protein and a substrate of Src kinase, can bind with c-Cbl. This interaction promotes osteoblast differentiation by inhibiting c-Cbl-induced mTOR degradation while downregulating RANKL expression to inhibit osteoclast differentiation (Yang et al., 2024). However, further research into the detailed mechanisms and regulatory networks of Cbl in osteogenic differentiation is necessary, as is further investigation into the signaling pathways and transcription factors that regulate OB differentiation and survival.

#### 2.2.4 WWP2

WWP2 is a member of the NEDD4 family, featuring an N-terminal WW domain and a C-terminal HECT domain. The WW domain can interact with multiple proline-rich sequences in proteins, while the HECT domain is responsible for linking WWP2 to target proteins and catalyzing the conjugation reaction (Jiang H et al., 2019; Jiang et al., 2022). WWP2 enhances the transactivation of RUNX2 through non-proteolytic monoubiquitination (Figure 3), playing an active role in osteogenesis (Zhu et al., 2017; Mokuda et al., 2019). Concurrently, studies on the interaction between WWP2 and Goosecid (GSC) reveal that the lack of the SOX9-Goosecoid signaling pathway in chondrocytes leads to severe craniofacial abnormalities and truncation of the torso in mice due to targeted disruption of WWP2 (Zou et al., 2011). Unlike WWP1, WWP2 catalyzes the monoubiquitination, rather than polyubiquitination, of RUNX2, thereby enhancing its transcriptional activity and osteogenic function (Zhu et al., 2017; Wang et al., 2024). Therefore, WWP2-mediated ubiquitination of RUNX2 is positively correlated with RUNX2 activity and bone development.In summary, WWP2 positively influences the process of osteogenic differentiation by regulating Runx2. These regulatory mechanisms are crucial for maintaining normal physiological functions of bones and preventing bone diseases.

#### 2.2.5 MDM2

The E3 ubiquitin ligase MDM2 is a member of the RING finger protein family, well-known for ubiquitinating the target protein p53 (Leslie et al., 2015). It regulates cell growth, DNA repair, oxidative stress, and apoptosis through promoting the degradation of p53 (Hauck et al., 2017). The crucial role of MDM2 in the differentiation of BMSCs to OBs has been confirmed (Chen et al., 2012; Chen et al., 2013; Daniele et al., 2019). Initially, the knockout of the Bre gene in mouse bone marrow stromal cells significantly reduced the expression of osteogenic marker genes, alkaline phosphatase activity, and mineralization capacity, thereby activating the p53 signaling pathway, as evidenced by increased expression of P53, p21, and MDM2 (Jin et al., 2017). As an upstream regulator of p53 in the DNA damage response, MDM2 can inhibit the activity of p53 by binding to it and promoting its ubiquitin-mediated degradation, thereby facilitating osteogenic differentiation (Wang et al., 2011) (Figure 3). Subsequently, p53 negatively regulates the differentiation of MSCs by downregulating the expression levels of the key transcription factors involved in early osteogenesis, Osx and Runx2 (Velletri et al., 2016). P53 acts as an inhibitor in osteogenic differentiation, while the negative regulatory effect of MDM2 can mitigate the inhibitory impact of p53, thus promoting osteogenic differentiation. Further research indicates that MDM2 may co-regulate with p53 in controlling the genes of osteocalcin during OBs differentiation (Chen et al., 2012). In summary, MDM2 collaboratively regulates OB differentiation with p53, playing a positive regulatory role in this process. The function of MDM2 may involve interactions with other regulatory factors. Its mechanisms in bone-related diseases, interactions with other signaling pathways, and *in vivo* mechanisms require further investigation.

## 3 The role of E3 ubiquitin ligase in bone-related diseases resource identification initiative

E3 ubiquitin ligases play a pivotal role in regulating bone formation and bone metabolism through their involvement in signaling pathways and transcription factors. Abnormal expression or dysfunction of E3 ubiquitin ligases may lead to bone-related diseases such as osteoporosis, osteoarthritis, bone tumors, and ankylosing spondylitis. Bone tissue is an organ distributed throughout the body, rendering it susceptible to pathological changes that may affect other organs (Chen et al., 2023). Consequently, E3 ubiquitin ligase represents a potential therapeutic target for bone-related diseases and warrants investigation into its regulatory mechanism and pathway of action, with the objective of developing new drugs and intervention strategies.

### 3.1 Osteoporosis

Osteoporosis is considered a systemic disease characterized by reduced bone mass and deterioration of bone microarchitecture, leading to increased bone fragility and a higher risk of fractures (Oei et al., 2016; Peng et al., 2018; Gu et al., 2020). Bone metabolic balance is regulated by the processes of bone formation and bone resorption (Appelman-Dijkstra and Papapoulos, 2015). Disruption of this balance results in osteoporosis. Osteoporosis is a complex multifactorial disease associated with various risk factors and medical conditions, with reduced osteogenic differentiation of BMSCs considered a fundamental pathogenic mechanism (Gu et al., 2020; Li et al., 2021). Additionally, the pathogenesis of osteoporosis is closely related to inflammation, hormones, oxidative stress, mitochondrial function, microRNAs (miRNAs), and medications (Iantomasi et al., 2023). E3 ubiquitin ligases play an indispensable role in this context.In terms of inflammation, studies have shown that the proteasome inhibitor bortezomib accelerates the degradation of the homologous receptor c-Fms of macrophage colony-stimulating factor (M-CSF), preventing lipopolysaccharide (LPS)-induced inflammatory bone resorption due to reduced numbers of c-Fms positive osteoclasts. Proteasome inhibitors accelerating the proteolysis of c-Fms protein could be a therapeutic option for inflammation-induced bone loss (Lee et al., 2017). Additionally, studies have found that the proteasome system inhibitor Withaferin A reduces Smurf2 expression, preventing the degradation of RunX2 and related Smad proteins, promoting osteoblast survival, and inhibiting inflammatory cytokines, suggesting that Withaferin A is a potential target for osteoporosis treatment (Khedgikar et al., 2013). Furthermore, octyl itaconate inhibits inflammation and osteoclastogenesis by inhibiting the E3 ubiquitin ligase Hrd1 and activating Nrf2 signaling, demonstrating potential application value in the treatment and prevention of osteoporosis (Sun et al., 2019). In terms of hormones, studies have found that carnosic acid (CA) directly binds to the ligand-binding domain of estrogen-related receptor alpha (ERRα), significantly promoting its ubiquitination and proteasomal degradation. Research suggests that CA can serve as a lead compound for drug control of osteoporosis. TP53INP2 is a target of the tumor suppressor gene p53, which promotes degradation by recruiting ubiquitinated substrates to autophagosomes. Regarding oxidative stress, the downregulation of TP53INP2 induced by oxidative stress inhibits the osteogenic differentiation of BMSCs in osteoporosis, mediated through autophagic degradation (Yang et al., 2021). In mitochondrial function, studies have found that 5,7-dimethoxyflavone (DMF), a natural compound, inhibits osteoporosis by improving protein turnover and mitochondrial function (Kim and Hwang, 2020). In terms of microRNAs, studies have found that miR-708-5p and miR-708-3p jointly accelerate the progression of osteoporosis through different but complementary mechanisms. This synergistic effect accelerates the decline in bone density and degradation of bone tissue, thereby advancing osteoporosis progression. The findings suggest that miR-708-5p and miR-708-3p could be targets for the prevention and treatment of osteoporosis (Wang R et al., 2020). Regarding medication, research has found that patients taking the antidepressant clomipramine have an increased risk of osteoporotic fractures. Clomipramine promotes osteoclastogenesis by upregulating the expression of the E3 ligase Itch, leading to osteoporosis. Clomipramine-induced bone loss through enhanced Itch-mediated osteoclastogenesis can be blocked by zoledronic acid. The results indicate that zoledronic acid could be a targeted drug for the prevention and treatment of osteoporosis (Li et al., 2017). Additionally, miR-216a significantly promotes the osteogenic differentiation of hAMSCs *in vitro*, counteracting the inhibitory effect of dexamethasone on osteogenesis and promoting bone formation *in vivo*. This suggests that miR-216a could be a novel therapeutic agent for the prevention and treatment of osteoporosis and other bone metabolic diseases (Li H et al., 2015). Osteoporosis presents with diverse clinical manifestations and can lead to severe health issues. The complex and varied pathogenic mechanisms interact to result in osteoporosis. Although some therapeutic approaches have made clinical advances, a deeper understanding of the interactions among these mechanisms is needed to develop more effective treatment strategies. Future research should focus on the comprehensiveness and specificity of pathogenic mechanisms and how these can be translated into targeted therapies.

### 3.2 Osteoarthritis

Osteoarthritis is a common degenerative joint disease, primarily characterized by the degradation of articular cartilage and joint inflammation (Carpintero-Fernandez et al., 2020). Initially, E3 ubiquitin ligases play a crucial role in the pathogenesis of osteoarthritis by regulating the metabolism of articular cartilage, inflammatory responses, and autophagy, thereby influencing the onset and progression of osteoarthritis. In terms of cartilage metabolism, studies have established a murine model of osteoarthritis by destabilizing the medial meniscus, finding that Itch degrades JAG1 via ubiquitination, inhibiting the activation of the Notch1 pathway, thereby reducing endotoxin-induced chondrocyte damage and osteoarthritic articular cartilage damage, offering new strategies for osteoarthritis treatment (Qi et al., 2022). Studies have found that activating the Nrf2 signaling pathway can inhibit the E3 ubiquitin ligase ZNF598, reducing the degeneration of the cartilage endplate (Huang B et al., 2023). Additionally, research has shown that the E3 ubiquitin ligase RNF125 can achieve therapeutic effects on osteoarthritis by inhibiting the Wnt/β-catenin signaling pathway (Lv et al., 2023). Regarding inflammatory responses, an increase in pro-inflammatory macrophages has been observed in joints with post-traumatic osteoarthritis (PTOA) in mice. Researchers using a macrophage Itch-deficient mouse model have found that the negative regulatory factor Itch inhibits pro-inflammatory polarization of macrophages, thereby limiting the progression of PTOA (Lin et al., 2021). Studies have found that the E3 ubiquitin ligase Trim14 may lead to chronic inflammatory pain by degrading IκBα, suggesting that Trim14 could be a new therapeutic target for chronic inflammatory pain (Niu et al., 2024). In the process of autophagy, RNA sequencing has been used to study the differential expression of E3 ubiquitin ligase genes in the cartilage of normal individuals and osteoarthritis patients. The results show that the expression of HECTD1 is significantly downregulated in the cartilage of osteoarthritis patients compared to normal cartilage, thus exacerbating the onset of surgically and aging-induced osteoarthritis (Liao et al., 2023). Simultaneously, the regulation of E3 ubiquitin ligases may become a new strategy for treating osteoarthritis, primarily by affecting chondrocytes and the state of cartilage. For instance, promoting the expression of FBXW7 can alleviate the symptoms of osteoarthritis (Zhang et al., 2022). Recent research has found that selenium (Se) intake can enhance antioxidant capacity and reduce inflammation, thereby decreasing cartilage loss and benefiting osteoarthritis (Cheng et al., 2024). E3 ubiquitin ligases play a key role in the pathological process of osteoarthritis, and their regulation may become a new strategy for treating osteoarthritis. Current research provides us with a preliminary understanding of the potential therapeutic role of E3 ubiquitin ligases; however, further studies are needed to reveal their detailed mechanisms of action and develop safe and effective treatments. Future research should focus on the development of targeted drugs for E3 ubiquitin ligases and their clinical applications to improve the quality of life for osteoarthritis patients.

### 3.3 Osteosarcoma

Osteosarcoma is a common malignant bone tumor in adolescents, characterized by lung metastasis and high mortality rates (Chang et al., 2021). The erosion and dissolution of cortical bone by tumor tissues cause pain in the affected areas, with prominent symptoms in patients. Osteosarcoma is highly heterogeneous, and its pathogenesis remains unclear.Increasing research indicates that E3 ubiquitin ligases play a significant role in the onset, invasion, metastasis, and targeting of osteosarcoma.Firstly, regarding the occurrence of osteosarcoma, M6A RNA methylation plays a crucial role in regulating the growth and progression of osteosarcoma cells, controlling tumor development through the regulation of USP22/RNF40 and histone ubiquitination (Yadav et al., 2022). Secondly, regarding the invasion and metastasis of osteosarcoma, studies have shown that overexpression of chromobox protein homolog 4 (CBX4) in osteosarcoma cell lines and tissues leads to the recruitment of GCN5 to the Runx2 promoter, upregulating Runx2 at the transcriptional level and thus promoting tumor metastasis. Casein kinase 1α (CK1α) promotes the phosphorylation of Cbx4 at T437, leading to its ubiquitination at K178 and K280 and subsequent ChIP degradation, thereby inhibiting cell migration and invasion through the suppression of Cbx4 (Wang X et al., 2020). This suggests that targeting the CK1α/CBX4 axis may benefit patients with metastatic osteosarcoma. Regarding the treatment of osteosarcoma, studies have found that Cullin 4b (CUL4B), a key member of the Cullin-RING E3 ligase, has been shown to interact with DNA damage-binding protein 1 (DDB1) and Cullin-4-associated factor 13 in human osteosarcoma cells to form an E3 complex that degrades the tumor suppressor PTEN, thereby promoting cell growth. Additionally, the 3′-UTR of CUL4B is a target of microRNA-300, maintaining PTEN stability. Furthermore, studies have found that TRIM22 regulates the proliferation and metastasis of osteosarcoma (OS) cells by promoting NRF2 degradation and activating the ROS/AMPK/mTOR/autophagy signaling pathway (Liu W et al., 2022). This indicates that the TRIM22/NRF2 pathway could be a promising target for osteosarcoma treatment. Moreover, research has shown that the targeted drug TSC01131 can disrupt the stability of the CRL4B^DCAF13^ E3 ligase, significantly inhibiting the growth of osteosarcoma cells (Chen et al., 2018). Other studies have found that geranylgeranylacetone (GGA) promotes the degradation of protein arginine methyltransferase 1 (PRMT1) via the Hsp70-Chip-mediated proteasomal pathway, thereby inducing Fas-triggered apoptosis. This suggests that GGA could be a targeted therapeutic drug for osteosarcoma (Chen et al., 2017). Additionally, research has identified the Rab22a-neoF1 fusion protein as an important target for lung metastasis of osteosarcoma. These findings reveal the lysosomal degradation mechanism of Rab22a-neoF1 fusion protein and suggest that Sorafenib and Regorafenib could be effective drugs for cancer patients expressing the RAB22A-NeoF1 fusion gene. In recent years, research on E3 ubiquitin ligases has gradually become a new strategy for treating osteosarcoma (Zeng et al., 2023). However, most current studies are still limited to animal experiments, and corresponding clinical research remains insufficient. Future research should aim to conduct more clinical trials.Although existing studies have shown the importance of E3 ubiquitin ligases in bone tumors, there are still many limitations and deficiencies. Future research needs to further investigate the functions and regulatory mechanisms of E3 ubiquitin ligases in osteosarcoma, particularly their interactions and coordinated regulation with other signaling pathways, and verify their efficacy and safety in clinical treatment.

### 3.4 Ankylosing spondylitis

Ankylosing spondylitis is characterized by chronic inflammation and pathological new bone formation in the sacroiliac joints and spine, primarily affecting the spine and ligaments, leading to arthritis and joint stiffness. Although the exact etiology of ankylosing spondylitis remains unclear, studies have shown that E3 ubiquitin ligases play a crucial role in its pathogenesis. Firstly, BMSCs from patients with ankylosing spondylitis exhibit abnormal angiogenic capabilities; increased expression of the SMAD-specific E3 ubiquitin ligase Smurf2 in BMSCs is a primary cause of this anomaly. Smurf2 regulates angiogenesis by promoting the ubiquitination and degradation of Pentraxin 3 (PTX3), highlighting the role of E3 ubiquitin ligases in the abnormal angiogenesis associated with ankylosing spondylitis pathology (Ma et al., 2021). Secondly, mucosa-associated lymphoid tissue lymphoma translocation protein 1 (MALT1) promotes the differentiation and immune response of CD4^+^ T cells, correlating with the severity and activity of inflammation in ankylosing spondylitis patients, suggesting that MALT1 may be a key regulator of the inflammatory response in this disease (Qin et al., 2021; Yuan et al., 2022). By regulating the expression of miR-125a-5p and TNFAIP3, the long non-coding RNA (lncRNA) MEG3 affects osteogenic differentiation of mesenchymal stem cells; overexpression of MEG3 and TNFAIP3 or inhibition of miR-125a-5p can suppress the osteogenic differentiation of BMSCs, offering new strategies for the treatment of ankylosing spondylitis (Liu C et al., 2023). Recent studies have identified circ-0110,634 as a target of triptolide, which, by dose-dependently inhibiting the extracellular transport of CIRC-0110634, can alleviate the burden on patients with ankylosing spondylitis (Ji et al., 2022). In summary, E3 ubiquitin ligases play a significant role in the pathogenesis of ankylosing spondylitis, participating not only in the regulation of inflammatory responses but also in osteogenic differentiation processes. Research on Smurf2, MALT1, and other related molecules has provided new biomarkers and therapeutic targets. However, the specific role of E3 ubiquitin ligases in the immune regulation and bone metabolism of ankylosing spondylitis still requires further study. Future research may focus on the specific regulatory roles of E3 ubiquitin ligases and explore their potential as therapeutic targets.

## 4 Conclusion and perspectives

Ubiquitin-proteasome system (UPS) is one of the primary pathways for protein degradation within cells (Meyer-Schwesinger, 2019). E3 ubiquitin ligases play a crucial role in the UPS by regulating the stability and function of target proteins through ubiquitination. The E3 ligase specifically recognizes and binds to the target protein, then transfers activated ubiquitin from the E2 ubiquitin-conjugating enzyme to the target protein, typically attaching it to the lysine residues of the target protein. By repeating this process multiple times, the target protein can be modified with several ubiquitin molecules, forming a polyubiquitin chain, which serves as a signal for proteasomal recognition and degradation. The recognition mechanisms of E3 ubiquitin ligases are diverse. E3 ubiquitin ligases selectively bind to target proteins by recognizing specific amino acid sequences within substrate proteins, which are typically referred to as degradation signals (Mashahreh et al., 2022). E3 ubiquitin ligases can also achieve substrate-specific binding by recognizing specific domains or three-dimensional conformations of the substrate proteins (Sluimer and Distel, 2018). Additionally, E3 ubiquitin ligases can also selectively bind to substrates by recognizing phosphorylation sites on the substrate proteins (Zhao Y et al., 2011). Moreover, some E3 ubiquitin ligases recognize and bind to substrate proteins through accessory proteins, such as adaptors or scaffold proteins (Akopian et al., 2022). Adaptor proteins typically have specific substrate-binding domains that can bridge the E3 ligase and the substrate protein. Through this multi-layered collaboration, E1, E2, and E3 enzymes collectively ensure the precision and specificity of the ubiquitination process, thereby regulating the dynamic balance of intracellular proteins.

E3 ubiquitin ligases also play a crucial role in osteogenic differentiation and bone-related diseases. They participate in regulating protein degradation, impacting osteogenic differentiation and the health of bone tissue (Sévère et al., 2013). In normal physiological processes, E3 ubiquitin ligases regulate key osteogenesis-related proteins through ubiquitination, modulating their stability and function to maintain normal bone metabolism and function. For example, the E3 ligase RNF185 negatively regulates the Wnt signaling pathway by degrading Dishevelled-2 (Dvl2) protein, thereby inhibiting osteogenic differentiation. Another E3 ligase, Itch, negatively regulates the bone morphogenetic protein (BMP) signaling pathway by promoting the degradation of Smad1, inhibiting the differentiation of osteoblasts. Conversely, the E3 ligase WWP2 positively regulates osteogenic differentiation by enhancing Runx2 activity through monoubiquitination. In the study of bone-related diseases, abnormal expression or dysfunction of E3 ubiquitin ligases is closely associated with the development of these diseases. For instance, the abnormal expression of E3 ubiquitin ligases in osteoporosis may lead to decreased bone density and increased risk of fractures. Research indicates that E3 ubiquitin ligases are involved in regulating osteoblast differentiation and bone formation, such as OTUB1 promoting bone formation by stabilizing FGFR2, and Parkin inhibiting RANKL-induced osteoclastogenesis. Therefore, in-depth research into the mechanisms of E3 ubiquitin ligases in osteogenesis and bone-related diseases is crucial for understanding the pathogenesis, prevention, diagnosis, and treatment of these diseases.

Although there is some understanding of the role of E3 ubiquitin ligases in osteogenesis, many gaps remain. Current research typically focuses on a few E3 ubiquitin ligases, but the E3 ubiquitin ligase family has many members, and the functions and roles of many other members in osteogenesis remain unknown. This diversity and specificity of functions have not been fully explored, possibly hiding new regulatory mechanisms. Additionally, although it is known that E3 ubiquitin ligases regulate key osteogenic signaling pathways and transcription factors through ubiquitination, their specific regulatory networks and interactions with other biological processes are still unclear. For example, it is not fully understood how E3 ubiquitin ligases coordinate with other ubiquitination-related enzymes, such as E1 ubiquitin-activating enzymes and E2 ubiquitin-conjugating enzymes, and how they impact the overall function of the ubiquitin-proteasome system. E3 ubiquitin ligases have shown potential roles in bone-related diseases in animal models. Currently, the role of E3 ubiquitin ligases in diseases such as osteoporosis, osteoarthritis, and osteosarcoma can be studied by constructing transgenic mouse models (Ying et al., 2016; Li et al., 2017; Mokuda et al., 2019). However, the direct relevance and clinical significance of these findings in humans are still uncertain. More clinical studies are needed to validate these potential mechanisms and explore their applications in osteoporosis, osteoarthritis, osteosarcoma, ankylosing spondylitis, and other skeletal diseases.

In future clinical research, in addition to studying the role of E3 ubiquitin ligases in osteoporosis, particularly their regulation of OB function. It is also necessary to explore the specific mechanisms of E3 ubiquitin ligases in cartilage degeneration and osteoarthritis. Concurrently, research should examine the role of E3 ubiquitin ligases in the proliferation and metastasis of osteosarcoma cells. Furthermore, the impact of E3 ubiquitin ligases on spinal inflammation and skeletal structure changes warrants investigation. Initially, as the E3 ubiquitin ligase family comprises hundreds of members, future studies should aim to identify more E3 ubiquitin ligases involved in the osteogenesis process. This includes employing high-throughput screening techniques to explore the specific roles of these enzymes in osteoblast differentiation and activity. Existing technologies, such as high-throughput screening and computational virtual screening, can be used to identify more small molecule modulators of E3 ubiquitin ligases. Simultaneously, further exploration of the structure of E3 ubiquitin ligases and their mechanisms of action on substrates will help identify more potential drug-binding sites. Additionally, understanding how E3 ubiquitin ligases precisely regulate osteogenesis-related signaling pathways is still in the preliminary stages. The application of more advanced biochemical and molecular biology techniques, such as single-cell sequencing and proteomic analysis, will help reveal the mechanisms of action of E3 ubiquitin ligases at the single-cell level (Jordan et al., 2023). Given the complex mechanisms of bone-related diseases, existing treatments are limited to symptom improvement and cannot prevent the disease course. Currently, designing targeted drugs for specific E3 ubiquitin ligases is considered a new strategy for treating these diseases. Research on targeted drugs for E3 ubiquitin ligases is currently limited. Using CRISPR/Cas9 and other gene editing technologies, specific E3 ubiquitin ligase genes can be precisely knocked out or knocked in (Tang et al., 2018). Concurrently, the development of small molecule inhibitors or activators specific to E3 ubiquitin ligases could serve as potential therapeutic drugs. Finally, most research is still in the *in vitro* and animal experimental stages. Translating these findings into clinical applications is a critical direction for future research. In the future, specific small molecule inhibitors or activators of E3 ubiquitin ligases can be developed and functionally validated both *in vivo* and *in vitro*. Techniques such as RNA interference (RNAi), quantitative real-time PCR (qPCR), and co-immunoprecipitation (Co-IP) can be used to study the signaling pathways and protein interactions of E3 ubiquitin ligases. By collaborating with clinicians and drug developers, the efficacy and safety of E3 ubiquitin ligase inhibitors or activators in treating osteoporosis, osteoarthritis, osteosarcoma, ankylosing spondylitis, and other skeletal diseases can be evaluated. Specifically, personalized treatment plans can be developed based on the expression patterns of E3 ubiquitin ligases in patients to enhance therapeutic efficacy. By detecting biomarkers associated with E3 ubiquitin ligases, early diagnosis and intervention of bone-related diseases can be achieved. Combining E3 ubiquitin ligase modulators with existing bone formation-promoting drugs can enhance therapeutic effects and improve patient prognosis.
